# Early-life mercury exposure and allergic diseases in childhood: systematic review and meta-analysis

**DOI:** 10.3389/fped.2026.1776559

**Published:** 2026-04-09

**Authors:** Qiming Liang, Maosong Liu, Hongliu Mao

**Affiliations:** Department of Pediatrics, Baoshan Branch of Renji Hospital Affiliated to Shanghai Jiao Tong University School of Medicine, Shanghai, China

**Keywords:** allergic diseases, allergic rhinitis, asthma, atopic dermatitis, children, eczema, mercury

## Abstract

**Background:**

Mercury is a persistent environmental contaminant that can cross the placenta and accumulate in fetal tissues. While its neurotoxic effects are well established, its potential association with allergic diseases in children remains unclear. Understanding how early-life mercury exposure relates to the burden of allergic conditions is important for child health and environmental risk assessment.

**Methods:**

We conducted a systematic review and meta-analysis of observational studies reporting allergic outcomes in children with measured mercury exposure. PubMed and Embase were searched from inception to October 2025. Studies were eligible if they assessed prenatal or postnatal mercury exposure and reported at least one allergic outcome. Random-effects meta-analysis was used to estimate pooled prevalence of allergic outcomes reported in studies.

**Results:**

Sixteen studies were included. The pooled prevalence among mercury-exposed children was 6.2% (95% CI, 1.8–18.8%) for asthma, 18.6% (95% CI, 9.0–34.5%) for atopic dermatitis, 14.4% (95% CI, 3.2–46.2%) for eczema, 15.8% (95% CI, 3.3–50.9%) for allergic rhinitis, and 22.3% (95% CI, 13.2–35.2%) for wheezing. Heterogeneity was high (*I*² > 90%). Studies from East Asia, which reported higher mercury exposure and fish intake, showed greater allergic disease proportions than European cohorts.

**Conclusions:**

Allergic diseases were frequent among populations in which mercury exposure was assessed, with regional and exposure-timing differences contributing to heterogeneity. The findings highlight the need for harmonised, prospective studies to clarify the role of mercury exposure in childhood allergic disease development.

## Introduction

The prevalence of allergic diseases has increased worldwide, particularly among children and adolescents (1). These conditions, including asthma, atopic dermatitis, allergic rhinitis, and food allergy, substantially affect quality of life and long-term health. The rapid rise in prevalence over recent decades cannot be explained by genetic factors alone and points to the influence of environmental exposures during critical periods of immune development. Accordingly, growing research (2, 3) has focused on early-life environmental determinants of allergic disease, including exposure to air pollutants, endocrine-disrupting chemicals, and heavy metals. Among these, mercury has emerged as a contaminant of particular concern.

Mercury is a toxic heavy metal that remains widespread in the environment. Human exposure occurs primarily through the consumption of contaminated fish and seafood (4), inhalation of mercury vapours, and contact with mercury-containing products. Methylmercury, the most toxic organic form, readily crosses the placental barrier (4) and accumulates in fetal tissues (4), raising concern about its developmental and immunological effects. Children are particularly vulnerable because of their higher exposure relative to body weight, immature detoxification capacity, and ongoing immune system maturation. Despite international efforts to reduce mercury emissions, it remains a persistent global contaminant.

There are plausible biological mechanisms linking mercury exposure to allergic diseases. Experimental studies (5, 6) have shown that mercury compounds promote oxidative stress, disrupt antioxidant defence systems, and alter cytokine profiles. Low-dose exposure in animal models enhances T helper type 2 immune responses, increases immunoglobulin E production, and elevates interleukin-4 and interleukin-13 expression, mediators central to allergic inflammation (7). Mercury can also activate mast cells and impair regulatory T-cell function, resulting in immune imbalance and enhanced sensitisation (8, 9). These mechanisms suggest that mercury may act as an immune adjuvant, amplifying allergic responses in genetically or environmentally predisposed individuals.

In addition to immunological pathways, mercury can influence metabolic and inflammatory processes (10) that may indirectly contribute to allergic disease. Methylmercury disrupts mitochondrial function, increases lipid peroxidation, and impairs antioxidant enzyme activity, leading to systemic inflammation and endothelial dysfunction (11). Mercury exposure has also been associated with altered glucose metabolism and increased insulin resistance, mechanisms that overlap with inflammatory pathways implicated in atopy (12). The convergence of these mechanisms underscores mercury's potential to affect immune regulation and tissue inflammation during early development.

Despite the biological plausibility of these pathways, epidemiological evidence linking mercury exposure to allergic outcomes in children remains limited and inconsistent. Observational studies have reported divergent findings. This inconsistency may reflect, in part, substantial methodological heterogeneity across studies, including variation in the biomarkers used to assess exposure (blood, urine, or hair mercury), differences in the exposure windows examined (prenatal maternal exposure versus postnatal childhood exposure), and variation in the allergic outcomes assessed. Despite accumulating cohort data, no prior quantitative synthesis has specifically summarized allergic disease outcomes among children with biomarker-measured mercury exposure. A structured synthesis is therefore warranted to clarify patterns across exposure windows and populations.

Accordingly, we conducted a systematic review and meta-analysis to synthesize the reported frequency and distribution of allergic diseases among children with measured mercury exposure.

## Method

### Search strategy

We searched MEDLINE (via PubMed), Embase and Web of Science from database inception to 12 October 2025 to identify observational studies investigating the relationship between mercury exposure and allergic diseases in children. The search strategy combined controlled vocabulary (MeSH terms) and free-text keywords related to mercury, methylmercury, children, and allergic diseases (including asthma, eczema, atopic dermatitis, allergic rhinitis, food allergy, and wheezing). This report follows the Preferred Reporting Items for Systematic Reviews and Meta-analyses (PRISMA) guideline (Supplementary Table S1).

### Study selection

We included original studies that: (1) involved participants aged ≤18 years or mother–child pairs; (2) measured mercury exposure using biological samples (e.g., cord blood, maternal blood, urine, or hair); (3) reported at least one allergic disease outcome, ascertained by physician diagnosis, validated questionnaire, or skin-prick testing; and (4) provided extractable data on the number or proportion of affected individuals. Eligible designs included prospective birth cohort studies, retrospective cohort studies, case control studies, and cross-sectional studies. We excluded non-english studies, animal studies, reviews, editorials, conference abstracts, and studies without primary data. All retrieved records were imported into EndNote and duplicates were removed. Two reviewers independently screened titles and abstracts for eligibility, followed by full-text review of potentially relevant studies. Discrepancies were resolved through discussion or consultation with a third reviewer. The selection process followed PRISMA recommendations and is summarised in a flow diagram, detailing the number of records identified, screened, excluded, and included in the final analysis.

### Data extraction

Two investigators independently extracted data from each eligible study using a standardised form. Extracted information included study characteristics (first author, publication year, country, study design, and sample size), participant characteristics (age of children and study population), and details of mercury exposure assessment. Exposure information included the biological matrix used for measurement (e.g., cord blood, maternal blood, child blood, urine, or hair), timing of exposure assessment (prenatal or postnatal), and reported mercury concentrations or exposure categories. Where available, we extracted covariates included in adjusted analyses, such as maternal allergy, parental smoking, fish intake, breastfeeding, and co-exposure to other environmental contaminants.

For each study, we extracted definitions and ascertainment methods for allergic outcomes—such as asthma, atopic dermatitis, eczema, allergic rhinitis, food allergy, and wheezing—along with the number and proportion of affected participants. When data were presented by subgroups (e.g., age), these were extracted separately. Disagreements were resolved by consensus.

### Quality assessment

We critically appraised all selected studies and formally assessed their quality by using a modification of the Newcastle-Ottawa scale (13) and the ROBINS-E framework for nonrandomized studies (14), in line with previous recommendations for quality assessment of observational studies (15). Studies scoring 7–9 points were classified as high quality, 4–6 points as moderate quality, and ≤3 points as low quality.

### Statistical analysis

Pooled proportions and 95% confidence intervals (CIs) were calculated using the inverse-variance method with DerSimonian–Laird estimators for between-study variance. Heterogeneity was quantified using *τ*² and *I*^2^ statistics.

When fewer than three studies were available for a given allergic outcome, quantitative pooling was deemed inappropriate because of limited precision and unstable variance estimates. These outcomes were therefore summarized descriptively. Subgroup analyses were conducted by type of allergic outcome (asthma, eczema, atopic dermatitis, wheezing, allergic rhinitis, food allergy) and, where data permitted, by age group (preschool vs. school-aged). All statistical analyses were conducted using R version 4.4.1 (R Foundation for Statistical Computing, Vienna, Austria), with significance levels set at *P* < 0.05 for all analyses.

## Result

**Figure 1:**
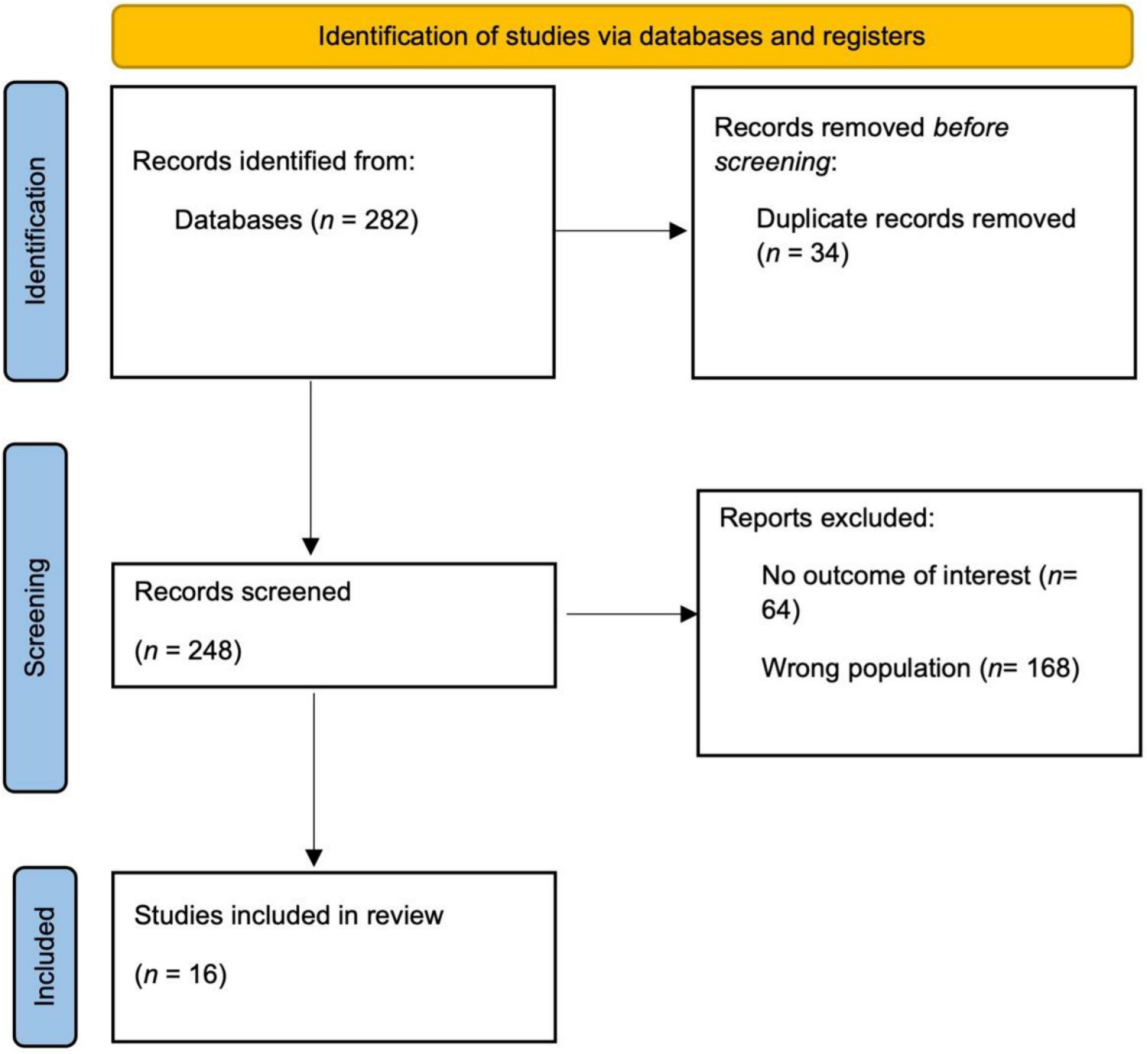
Prisma flow diagram.

Overall, 282 records were identified, and 34 duplicates were removed. After screening of titles and abstracts, 248 studies remained for full-text appraisal. Of those, 16 studies were eligible for inclusion (Figure 1).

Sixteen studies (16–31) encompassing diverse populations from Europe, Asia, and North America were included (Table 1). Most cohorts originated from East Asia (Korea and Japan), with additional studies from Spain, Greece, Poland, Germany, Taiwan, China, and the United States. Mercury exposure was assessed using cord blood, maternal blood, child blood, urine, or hair samples, reflecting both prenatal and postnatal exposure windows. Five (16, 19–21, 26) included studies reported information on breastfeeding practices or duration (Table 1), with three (16, 20, 21) further examining its role in relation to mercury exposure and allergic outcomes.

**Table 1:** Summary of included studies.

Included studies	Country	Measurement of Mercury	Mercury Exposure^a^	Key confounders/co-exposures	Breastfeeding information	Children age	Outcomes
Carrasco et al. (16)	Spain	Cord blood and children's hair	Geometric mean cord blood mercury: 8.2 μg/L; geometric mean of Hg in the 4-year old children's hair was 0.97 μg/g	15.9% of mothers reported active smoking during pregnancy, 60.1% reported passive smoking exposure, and 47.7% had a family history of allergic disease.	14.7% without breastfeeding, 44.3% with over 6-month breastfeeding	52.5 ± 2.3 months at the evaluation	Questionnaire; Wheezing 22.6%; Eczema 16.6%
							No significant association was reached.
Heinrich et al. (17)	Korea	Children's blood and urine	Mean blood mercury geometric means: 2.02 µg/L at 7–8 years, 1.79 µg/L at 9–10 years, and 1.96 µg/L at 11–12 years.	33% reported passive smoking exposure, and 7% had a family history of asthma.	Not reported	7–8 years old	Questionnaire; Hg at 7–8 y associated with asthma at 9–10 y (OR 1.6, 95% CI 1.2–2.1) and 11–12 y (OR 1.3, 95% CI 1.0–1.6).
Hon et al. (18)	China (HongKong)	Children's blood	Mean mercury concentration: 3.03 ± 2.33 µg/L	Not reported	Not reported	mean age 9.9 yr, s.d. 4.6 y	Physician diagnosis; no significant association was found
Jedrychowski et al. (19)	Poland	Cord blood, maternal blood at delivery	Mean cord blood mercury concentration: 8.8 µg/L (95% CI 8.1–9.5); maternal blood: 6.0 μg/L (95% CI: 5.4–6.7)	Maternal age 27.8 ± 3.4 years; cotinine and PAH adducts measured as co-exposures; smoking not reported	55.1% with breastfeeding exclusive over 3 months	5 years	Skin-prick testing; no significant association between cord blood mercury and atopy [RR 1.11 (0.68–1.80)]
Kampouri et al. (20)	Greece	Maternal blood and children's blood	Mean maternal blood mercury: 1.82 ± 1.30 µg/L; infant blood mercury: 1.02 ± 0.92 µg/L	69% parental allergy; 6% maternal smoking; socioeconomic and dietary data collected	88% breastfeeding between at 4 months	1 year	Physician diagnosis; asthma (*n* = 39/428) and food allergy (*n* = 33/449); no association with gestational mercury [fully adjusted OR 1.02 (95% CI 0.67–1.55)]
Kim et al. (22)	Korea	Children's blood	Mean mercury concentration: 2.4 ± 0.9 µg/L	2% parental allergy; no smoking data reported	Not reported	Not specified (school-aged children)	Skin-prick testing; blood mercury positively associated with allergic sensitisation [adjusted OR 1.6 (95% CI 1.1–2.3)]
Kim et al. (23)	Korea	Children's blood	Geometric mean blood mercury 2.02 µg/L at 7–8 years, 1.79 µg/L at 9–10 years, and 1.96 µg/L at 11–12 years	32% passive smoking; 2% maternal asthma; 2% paternal asthma	Not reported	7–12 years	Questionnaire; blood mercury at 7–8 years associated with asthma up to 9–10 years [OR 1.6 (95% CI 1.2–2.1)] and 11–12 years [OR 1.3 (95% CI 1.0–1.6)]; significant in Cox model [HR 1.4 (95% CI 1.1–1.8)]
Kim et al. (21)	Korea	Cord blood	Median mercury 7.2 µg/L (range 1.6–71.5 µg/L)	28.7% maternal allergic disease; smoking not reported	Not reported	60 months (range, 24.0–69.0 months)	Physician diagnosis; no significant association between cord blood mercury and atopic dermatitis [multivariable HR 0.98 (95% CI 0.73–1.32)]
				32% with exclusive breastfeeding during the first 6 month			
Lee et al. (25)	Korea	Maternal blood	Mean maternal mercury 3.31 ± 1.58 µg/L in early pregnancy and 3.07 ± 1.61 µg/L in late pregnancy	3.5% maternal allergy; smoking not reported	Not reported	6 months	Questionnaire; incidence rate of AD was 27.1% in 6-month-old-infants and 27.7% in boys and 26.3% in girls. Higher mercury exposure showed no significant association with the outcome [Q2 OR 1.02 (95% CI 0.66–1.57); Q3 OR 1.08 (95% CI 0.70–1.68); Q4 OR 0.95 (95% CI 0.59–1.52), reference = Q1^b^].
Lee et al. (24)	Korea	Urine	Geometric mean total urinary mercury 0.401 µg/L (3rd cycle) and 0.399 µg/L (4th cycle)	Not reported smoking and family history	Not reported	6-11 years	Questionnaire; urinary mercury positively associated with atopic dermatitis [cycle 3 OR 1.36 (95% CI 1.02–1.81), cycle 4 OR 1.21 (95% CI 0.64–2.29)] and with asthma after full adjustment
Miyake et al. (26)	Japan	Children's Hair	Children's hair mercury range 0.13–9.51 µg/g, median 1.38 µg/g (95th 3.74)	Maternal allergy 43.1%; paternal allergy 31.8%; maternal smoking during pregnancy 11.3%; maternal fish intake 48.7 ± 27.1 g/day	maternal age 30.2 ± 3.9 y	29 to 39 months	Questionnaire; no significant association between child hair mercury and wheeze [adjusted OR 1.09 (95% CI 0.90–1.31)] or eczema [adjusted OR 1.00 (95% CI 0.81–1.21)]
					76% with >= 6-month breastfeeding		
Miyazaki et al. (27)	Japan	Maternal blood	Mean ± SD 4.21 ± 2.49 µg/L [median 3.64 (IQR 2.54–5.20); range 0.18–58.80 µg/L]	Mean maternal age 31.0 years; mean fish intake 33.9 g/day; 10.8% had pre-pregnancy BMI ≥25 kg/m^2^; maternal history of dermatitis 15.5%, food allergy 4.8%, asthma 10.8%, allergic rhinitis 35.6%; 4.4% preterm birth (<37 wk)	Not reported	1–3 years	Questionnaire; atopic dermatitis 15.5%, food allergy 4.8%, asthma 10.8%, allergic rhinitis 35.6%; higher maternal Hg associated with increased risk of AD (aOR 1.10, 95% CI 1.02–1.18) and asthma (aOR 1.08, 95% CI 1.01–1.15)
Schäfer et al. (28)	Germany	Children's blood and urine	Blood Hg: I < 0.1 µg/L 2.1%; II 0.1–0.3 µg/L 5.3%; III 0.3–0.5 µg/L 2.3%; IV > 0.5 µg/L 2.7%. Urinary Hg: I < 0.2 µg/L 2.2%; II 0.25–0.4 µg/L 4.3%; III 0.4–1.0 µg/L 2.7%; IV > 1.0 µg/L 2.8%.	Parental history of eczema 6.2%; current household smoking 2.3%	Not reported	5-14 years	Physician diagnosis; atopic eczema in 56 of 2,200 children (2.6%)
Shin et al. (29)	Korea	Cord blood and children's blood	Geometric mean cord blood mercury 5.1 µg/L (Q1 3.8; Q2 5.1; Q3 6.9 µg/L). GM at 24 months 2.2 µg/L (Q1 1.6; Q2 2.1; Q3 3.0 µg/L); at 36 months 2.2 µg/L (Q1 1.5; Q2 2.1; Q3 2.8 µg/L).	19% of mothers consumed <1 serving of fish per week, 64% consumed ≥1 serving	Not reported	6–60 month	Questionnaire; cord blood Hg associated with AD at 12–24 months (aOR 1.1, 95% CI 1.0–1.2). Child blood Hg at 24 months associated with AD at 24–36 months (aOR 1.2, 95% CI 1.1–1.4) and 48–60 months (aOR 1.5, 95% CI 1.1–1.9). No significant associations at 36 months.
Wei et al. (30)	USA	Children's Blood	Mean blood mercury 0.68 ± 0.03 µg/L	Not reported	Not reported	1–19 years	Physician diagnosis; 15.37% with asthma and 3.26% with hay fever; the weighted prevalence of eczema in non-adults was 13.42%; When mercury concentrations were divided into tertiles (0.14–0.23, 0.24–0.61, and 0.62–12.9 µg/L), the crude odds ratios for eczema were 1.00 (reference), 0.96 (95% CI 0.65–1.41), and 0.80 (95% CI 0.52–1.25), respectively
Wu et al. (31)	USA	Children's Blood	Mean blood mercury concentration 2.71 ± 0.10 μg/L.	85.7% under Environmental tobacco exposure; 14.9% under prenatal maternal smoking exposure		2–15 years	Questionnaire; No significant associations were observed between blood mercury and allergic outcomes (adjusted OR 1.00, 95% CI 0.95–1.05). Adjusted ORs for mercury and allergic diseases were 0.96 (95% CI 0.87–1.05) for ages 2–5, 0.95 (95% CI 0.90–0.99) for 6–11, and 1.02 (95% CI 0.93–1.11) for 12–15 years

^a^ Blood/cord blood in µg/L, hair in µg/g, urine in µg/L.

^b^ Q1 1st quartile of each exposure concentration.

While exclusively breastfed infants demonstrated higher mercury concentrations in one cohort, suggesting a potential postnatal exposure pathway, adjustment for breastfeeding did not materially change prenatal exposure–outcome associations in other studies. But one study (16) reported that prolonged breastfeeding was independently associated with reduced wheezing risk. Interpretation of these findings is limited because information on breastfeeding practices was not consistently reported or included in adjusted analyses across studies.

### Risk-of-bias assessment

Of the sixteen included studies, nine (16, 19–21, 23–27, 29) (56%) were rated as high quality and seven (17, 18, 22, 28, 30, 31) (44%) as moderate quality based on Newcastle–Ottawa Scale. ROBINS-E assessments (Supplementary Table S2) indicated that most studies were at low risk of bias (16, 19–21, 24, 27) or had some concerns, with three studies judged to be at high overall risk (18, 22, 28) of bias. In the ROBINS E assessment, the domains that most frequently contributed to higher risk of bias were confounding, particularly incomplete adjustment for dietary factors such as fish intake, and variation in outcome ascertainment methods, including questionnaire based reporting and physician diagnosis.

## Asthma

**Figure 2:**
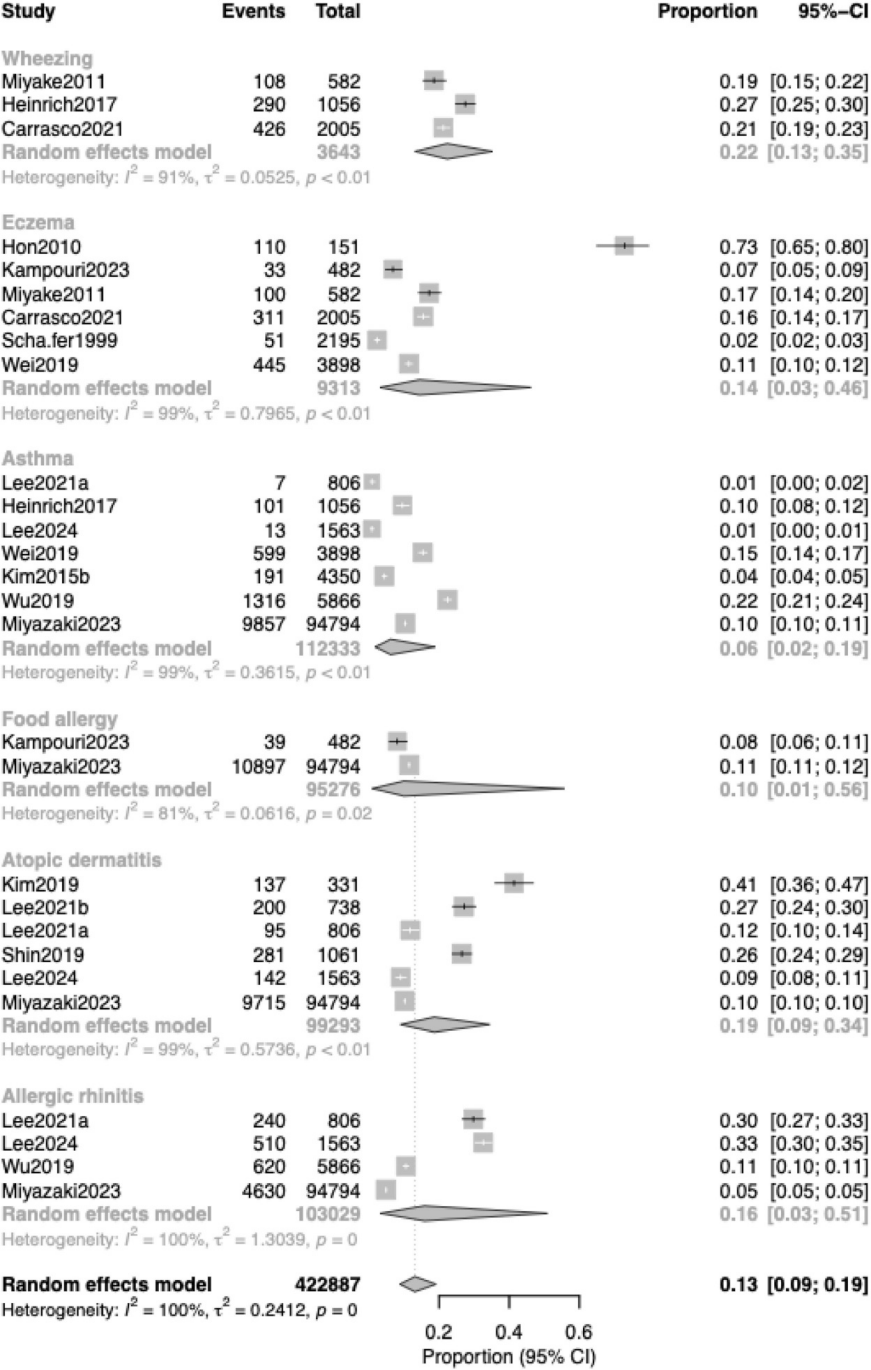
Pooled prevalence of allergic diseases in children according to disease type.

**Figure 3:**
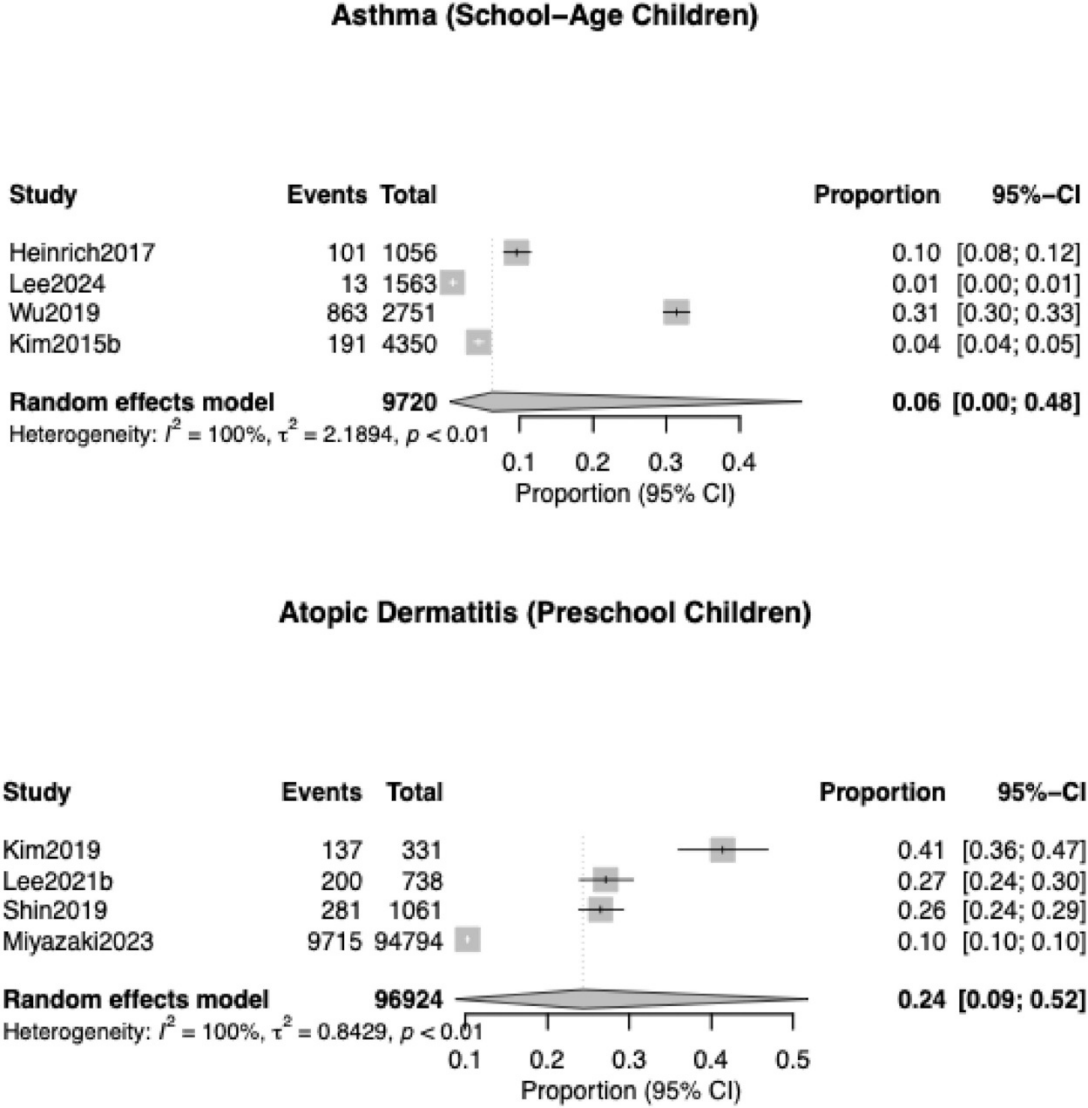
Subgroup analyses by age group for asthma and atopic dermatitis. Six studies examined eczema as an outcome distinct from atopic dermatitis. The pooled prevalence was 14.4% (95% CI, 3.2%–46.2%), with substantial heterogeneity (*I*² = 98.9%, *p* < 0.001).

Seven studies (20, 23–25, 27, 30, 31) (*n* = 9,720) reported asthma outcomes following exposure to mercury exposure. The pooled prevalence of asthma was 6.2% (95% CI, 1.8–18.8%), with substantial heterogeneity (*I*² = 99.5%, *p* < 0.001) (Figure 2). In analyses limited to school-aged children (Figure 3), the pooled prevalence was similar [6.3% (95% CI, 0.5–48.5), four studies]. Mercury exposure was primarily quantified through blood or serum biomarkers, with mean concentrations ranging from 0.36 µg/L in a low-exposure German cohort (Heinrich 2017) to approximately 3 µg/L in Korean studies (23, 24).

### Atopic dermatitis and eczema

Six studies (*n* = 96,924) assessed atopic dermatitis in relation to heavy-metal exposure. The pooled prevalence was 18.6% (95% CI, 9.0–34.5%) (Figure 2). Heterogeneity was high (*I*^2^ = 99.3%, *p* < 0.001). In preschool children, the pooled prevalence was higher at 24.3% (95% CI, 8.8%–51.8%) (Figure 3).

### Allergic rhinitis

Four studies reported allergic rhinitis outcomes. The pooled prevalence was 15.8% (95% CI, 3.3%–50.9%), with marked heterogeneity (*I*^2^ = 99.9%, *p* < 0.001) (Figure 2).

### Wheezing

Three studies evaluated wheezing symptoms. The pooled prevalence was 22.3% (95% CI, 13.2%–35.2%), the highest among all allergic phenotypes (Figure 2). Heterogeneity remained high (*I*^2^ = 90.8%, *p* < 0.001). In one cohort of preschool children, 22.6% experienced wheezing, yet both prenatal and postnatal mercury concentrations measured in cord blood and hair showed no association with symptom occurrence (16). Among school-aged participants (17), blood mercury concentrations were similarly unrelated to wheezing reported within the previous 12 months (adjusted OR 0.90, 95% CI 0.79–1.14). A separate prospective study (26) using children's hair samples also reported no relationship between mercury and wheezing (adjusted OR 1.09, 95% CI 0.90–1.31).

### Food allergy

Two studies assessed food allergy in relation to mercury exposure. In the Greek cohort (20), the prevalence of physician-diagnosed food allergy was 8.3% (39/467), with no significant association between gestational erythrocyte mercury and risk of food allergy after full adjustment (OR 1.02, 95% CI 0.67–1.55). In contrast, the Japanese cohort (27) (*n* = 94,794) reported 10,897 cases of food allergy (10.9%) within the first three years of life, and higher maternal blood mercury levels were associated with a small but statistically significant increase in risk (adjusted OR 1.03, 95% CI 1.00–1.05) after adjustment for selenium, manganese, and gestational week at sampling. Differences in exposure assessment, outcome definitions, adjustment strategies, and population characteristics between the two cohorts may contribute to the variation in reported findings.

## Discussion

This systematic review and meta-analysis provide a comprehensive synthesis of the reported prevalence of allergic diseases among children with measured mercury exposure. Across the included studies, the reported proportions of allergic outcomes such as asthma, atopic dermatitis, eczema, and wheezing varied widely across populations. Substantial heterogeneity was observed, reflecting differences in exposure assessment, study design, and geographic setting. Although this review does not allow causal inference, some consistent patterns emerged. Across the included studies, the reported proportions of allergic outcomes varied substantially between cohorts. Studies conducted in East Asian populations often reported higher proportions of allergic conditions than studies from Europe or North America. These differences should be interpreted cautiously. Variation in reported prevalence may reflect differences in underlying population risk, diagnostic practices, study design, dietary patterns, and co-exposure to other environmental factors, in addition to potential differences in mercury exposure levels.

The variation in allergic disease proportions observed across the included studies may suggest differences in both biological timing of exposure and age at outcome assessment. Mercury, particularly in its methylated form, is known to cross the placental barrier and accumulate in fetal tissues during gestation (32, 33). Cohorts that measured prenatal exposure, such as those from Japan and Korea (24, 27), reported higher proportions of allergic outcomes—particularly atopic dermatitis and wheezing—than those measuring postnatal or concurrent exposure (16, 17). This pattern is biologically plausible, as fetal and early postnatal periods are characterised by rapid immune differentiation, thymic development, and establishment of tolerance mechanisms (34). Disruption during these critical windows may alter T-helper cell balance and regulatory T-cell function, increasing susceptibility to later allergic responses (35).

Experimental and epidemiological studies support this developmental interpretation. Methylmercury exposure induces oxidative stress and activates nuclear factor-*κ*B and mitogen-activated protein kinase pathways, promoting T-helper type 2 cytokine dominance and enhancing interleukin-4 and immunoglobulin E production—key mediators in allergic inflammation (36, 37). Mercury also upregulates major histocompatibility complex class II molecules and impairs regulatory T-cell activity, amplifying antigen presentation and immune reactivity (7, 38, 39).These pathways align with the higher pooled proportions of atopic dermatitis and wheezing found in younger or preschool-aged cohorts in our review, suggesting that early exposure may prime immune dysregulation that becomes clinically evident within the first few years of life.

Across studies that included school-aged children, the pooled prevalence of asthma remained similar to that observed in the overall analysis. This consistency suggests that age alone does not fully explain the variation in asthma proportions reported across cohorts. Differences between studies are more likely related to study design, exposure assessment methods, and population characteristics rather than to developmental timing. The included cohorts varied in whether mercury exposure was measured in blood, urine, or maternal samples, and these methodological differences may have contributed to the heterogeneity observed. In addition, school-aged populations were often drawn from different regions, with varying dietary habits, background pollutant exposures, and diagnostic criteria for asthma. Together, these factors may account for the wide confidence intervals and high heterogeneity identified in the pooled estimates. The current evidence therefore indicates that while asthma prevalence among populations in which mercury exposure was assessed appears consistent across age groups, the strength of this observation remains limited by the variability in study methods and population contexts.

Nutritional context further modulates these relationships. Fish and seafood, the primary dietary sources of methylmercury, also provide omega-3 fatty acids, selenium, and vitamin D—nutrients with well-established anti-inflammatory and immunoregulatory properties. The coexistence of beneficial nutrients and toxic metals creates a “nutrient–toxin paradox” that may partly explain the regional variation observed in allergic outcomes. For instance, in European cohorts such as INMA (16), where fish consumption mainly involves low-mercury species and diets are rich in antioxidants and unsaturated fats, the reported proportions of eczema and asthma were lower than in East Asian cohorts with higher consumption of predatory fish and greater methylmercury burden (29, 27). This divergence suggests that the protective effects of omega-3 fatty acids may counterbalance the pro-oxidant and Th2-skewing effects of mercury in some populations. Conversely, in contexts where methylmercury exposure is high and intake of omega-3s or selenium is insufficient, mercury-induced oxidative stress and cytokine dysregulation may predominate, resulting in stronger Th2-mediated inflammation and higher prevalence of skin and airway allergic diseases. However, only a subset of the included studies accounted for dietary factors such as fish consumption or micronutrient intake. Consequently, the present synthesis cannot determine the extent to which nutritional factors modify the relationship between mercury exposure and allergic disease.

A further consideration is the role of familial and maternal allergic predisposition, which may modify the child's immune response to environmental exposures such as mercury. In several included cohorts, between one-quarter and one-third of mothers reported a personal history of allergic disease (16, 21, 27), reflecting the well-established heritability of atopic disorders. Maternal atopy can influence fetal immune development through both genetic and intrauterine mechanisms. Pregnant women with allergic disease often exhibit a T-helper type 2–dominant cytokine profile, with elevated interleukin-4 and immunoglobulin E levels, which may cross the placenta and shape the fetal immune milieu (40). Such maternal immune skewing could lower the threshold for environmental stimuli, including methylmercury-induced oxidative stress or antigen presentation, to provoke sensitisation in offspring. Evidence from animal and human studies suggests that mercury enhances Th2 cytokine production and reduces regulatory T-cell differentiation (41), potentially amplifying this inherited immune bias. The interaction between maternal atopy and prenatal toxicant exposure may therefore represent a synergistic pathway through which environmental and genetic factors converge to increase allergic susceptibility. This mechanism could partly account for the higher proportions of allergic outcomes observed in cohorts where maternal allergy prevalence was high.

## Strengths and limitations

This review integrates data across multiple continents and exposure windows, providing an overview of allergic disease prevalence among children with documented mercury exposure. The inclusion of both prenatal and postnatal measurements captures a broad developmental perspective. Nonetheless, several limitations should be acknowledged. The included studies differed substantially in exposure matrices, outcome definitions, and analytical methods, contributing to heterogeneity. Residual confounding from diet, socioeconomic status, and co-exposure to other environmental agents remains possible. Most included studies were observational and did not report comparative effect estimates, precluding evaluation of relative risk. Variation in disease ascertainment, including reliance on parental reporting in some studies, may also have introduced measurement bias. Although several studies reported breastfeeding practices, measurement was inconsistent and typically limited to duration, without direct assessment of mercury concentrations in breast milk. This limits the ability to disentangle breastfeeding as a potential exposure pathway from its established immuno protective effects. Outcome ascertainment also varied across studies. Some cohorts relied on physician diagnoses recorded in clinical assessments, whereas others used questionnaire-based reporting or objective measures such as skin-prick testing. Differences in outcome definition and ascertainment may have contributed to heterogeneity in the reported prevalence estimates. Mercury exposure was assessed using different biological matrices across studies. These biomarkers reflect different exposure windows and toxicokinetic properties. This variability in exposure assessment may have contributed to the heterogeneity observed across studies and limits direct comparison of exposure levels between cohorts. In addition, the limited number of studies within individual biomarker categories prevented stratified meta-analyses by exposure matrix or timing (prenatal versus postnatal).
